# Ti_12_C_68_: A stable *T*_*h*_-symmetry hollow cage

**DOI:** 10.1038/s41598-018-22381-y

**Published:** 2018-03-08

**Authors:** Ling-Yan Ai, Hui-Yan Zhao, Hong-Man Ma, Jing Wang, Ying Liu

**Affiliations:** 1 Department of Physics and Hebei Advanced Thin Film Laboratory, Hebei Normal University, Shijiazhuang, 050024 Hebei, China; 2 North China University of Science and Technology, Tangshan, 063000 China; 3 National Key Laboratory for Materials Simulation and Design, Beijing, 100083 China

## Abstract

A stable *T*_*h*_-symmetry Ti_12_C_68_ cage was systemically investigated using density functional theory. The structure of Ti_12_C_68_ is a hollow cage with twelve TiC_13_ subunit of three pentagons and one hexagon. The calculated frequencies are in the range 95.1 cm^−1^–1423.9 cm^−1^. There are no imaginary frequencies, showing its kinetic stability. *Ab initio* molecular dynamics simulations demonstrate that the topological structure of cage-like Ti_12_C_68_ cluster was well maintained when the effective temperature is up to 1139 K. The natural bond orbitals analysis shows that the *d* orbit of Ti atoms form four σ bonds with the neighboring four carbon atoms in each TiC_13_ subunit playing an important role in the cluster stability. The molecular frontier orbitals analysis indicates that Ti_12_C_68_ cage has a narrow HOMO-LUMO gap with metal-like property. It would be expected to enrich the species of hollow metal carbide clusters.

## Introduction

Recently, an exceptionally stable hollow molecule Sc_20_C_60_, named volleyballene^1^, has been proposed. Along with suggestion for Sc_20_C_60_, subsequent investigations resulted in the molecules of volleyball-like shape Y and La analogues^2^. Sc_20_C_60_ containing transition metals and carbon atoms belongs to the family of metal carbide clusters. Early famous metal carbide clusters, metallo-carbohedrenes (met-cars) ${M}_{{\rm{8}}}{C}_{{\rm{12}}}^{+}$, known as a unique class of cluster materials, which have been extensively investigated^3–5^. The ground-state structure of met-cars was once the center of debate. Early studies proposed for the containing a pentagonal dodecahedron structure similar to C_20_, the subsequent proposal that the isomers of ${{\rm{Ti}}}_{{\rm{8}}}{{\rm{C}}}_{{\rm{12}}}^{+}$ having different-symmetry structures with much lower energies^6–16^, have been reported. In particular, the inchoative Ti_8_C_12_ geometric structure has unique bonding properties that the d orbitals of Ti atoms play an important role in the cluster stability. Thus, theoretical investigations focus on an explanation for the electronic structure of novel isomers systems. Results exhibit diversities due to the existence of transition metal elements^11,14,15,17,18^. It is worth noting that the present structures of metal carbide clusters have been different with the originally established molecular model as alluded to above. Metal carbide clusters exhibit various geometric structure that determine their novel and rich physical and chemical properties^19–28^. Geometric configuration of each type met-cars cluster plays an important role in analyzing its characteristic and exploring the absorption mechanism of small molecules^29–31^.

Meanwhile, metal atoms^32–36^ and metal carbide cluster^37–39^ encapsulated inside different size fullerene cages form different kinds of endohedral metallofullerenes (EMF) which have attracted special attention owing to their unique structural, electronic, and magnetic properties. For example, encapsulating La atom into C_60_ fullerene cage^35^ was first discovered by Smalley *et al*. Later, dimetallofullerenes Ce_2_@C_80_^33^, Dy_2_@C_100_^34^, La_2_@C_80_^36^, metal carbide clusterfullerenes La_2_C_2_@C_s_(574)-C_102_^38^ and Y_2_C_2_@D_5_(8)-C_100_^39^ have been extensively studied. It is noteworthy that endohedral metallofullerenes enrich the family of metal carbide clusters and have promising applications in different fields such as materials science^40^ and medicine^41^. Up to now, the investigations of metal carbide clusters have become one of the highlights in nanoscience due to their exceptional stability and unique structures.

In the present work, we proposed a stable cage-like Ti_12_C_68_ structure and structural properties, and the stability of Ti_12_C_68_ cage has been systematically investigated within the density functional theory. Furthermore, the electronic properties and the natural bond orbitals analysis have been explored.

## Results and Discussion

**Figure 1:**
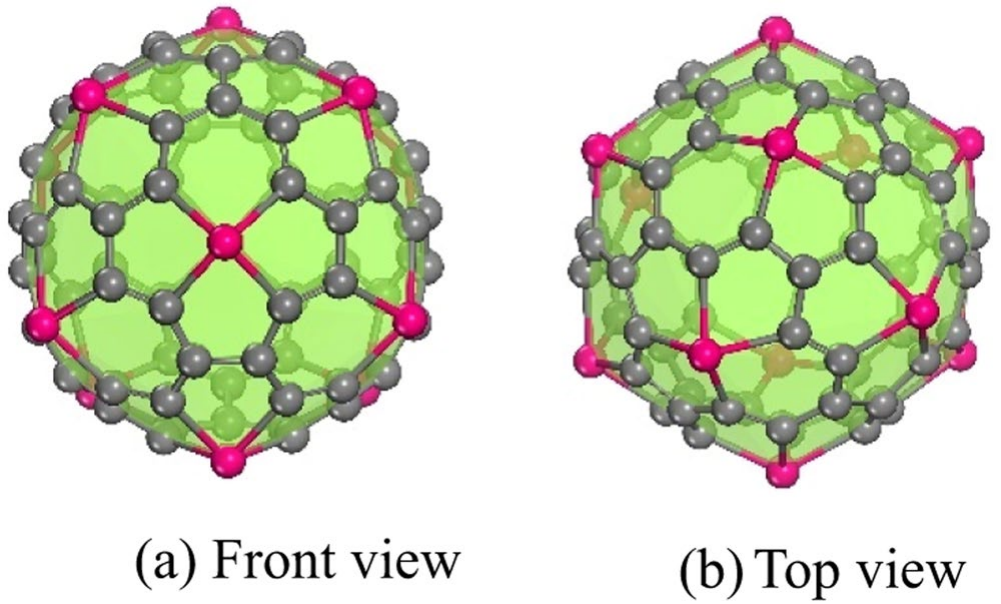
Front view (**a**) and top view (**b**) for cage-like Ti_12_C_68_ structure. Pink and gray spheres represent Ti and C atoms, respectively.

A schematic diagram of the optimized Ti_12_C_68_ structure is shown in Fig. 1. It is found to be a cage-like configuration with *T*_*h*_ symmetry, which contains twelve TiC_13_ subunits seamed together. Each TiC_13_ subunit, just as highlighting blue atoms constitute the unit in the left figure of Tab. 1, in which three pentagons share a single titanium atom with one hexagon. Each TiC_13_ subunit is bound to five neighbouring TiC_13_ subunits through C-C bonds. As seen from the Fig. 1 that the cage-like Ti_12_C_68_ is composed of 12 hexagon rings and 36 pentagonal rings and each of the rings contains one titanium atom. Titanium atoms occupy twelve unique positions and they have same coordination number.

In each TiC_13_ subunit, each Ti atom links four carbon atoms to form four Ti-C bonds. Ti-C bonds may be divided into two types. One type is in a hexagon denoted by ${d}_{\mathrm{Ti}-C}^{{\rm{1}}}$, another type is in a pentagon denoted by ${d}_{\mathrm{Ti}-C}^{{\rm{2}}}$. The pentagons and hexagons, and two neighbouring pentagons are connected by different C-C bonds which can be put into four categories, as shown in Table 1. As a result of bond-length equalization, such as 1, 3-butadiene, it leads C-C bond lengths lying between bond lengths of 1.54 Å (C–C bond) and 1.34 Å (C = C bond) in the optimized structure. The calculated properties of cage-like Ti_12_C_68_ are listed in Tab. 1 at the GGA/PBE, GGA/PW91 and GGA/BLYP levels, which gave the uniform structures and similar calculation results. In the following, analyses were treated within the GGA using the PBE exchange-correlation functional.

**Table 1:** Calculated properties for cage-like Ti_12_C_68_ structure at the GGA/PBE, GGA/PW91 and GGA/BLYP levels.

		Average bond lengths						Frequencies		*E*_b_	*E*_g_
		${{\boldsymbol{d}}}_{{\bf{Ti}}-{\bf{C}}}^{{\bf{1}}}$	${{\boldsymbol{d}}}_{{\bf{Ti}}-{\bf{C}}}^{{\bf{2}}}$	${{\boldsymbol{d}}}_{{\bf{C}}-{\bf{C}}}^{{\bf{1}}}$	${{\boldsymbol{d}}}_{{\bf{C}}-{\bf{C}}}^{{\bf{2}}}$	${{\boldsymbol{d}}}_{{\bf{C}}-{\bf{C}}}^{{\bf{3}}}$	${{\boldsymbol{d}}}_{{\bf{C}}-{\bf{C}}}^{{\bf{4}}}$	Lowest	Highest		
	PBE	2.153	2.112	1.398	1.428	1.444	1.441	95.5	1423.9	−6.86	0.05
	PW91	2.157	2.121	1.395	1.427	1.444	1.440	96.8	1410.4	−6.87	0.10
	BLYP	2.179	2.147	1.398	1.436	1.455	1.448	100.0	1360.3	−6.35	0.10

The average bond lengths, the lowest and highest vibrational frequency, binding energy per atom (*E*_b_), HOMO-LUMO gap (*E*_g_) are listed. The units of bond lengths, frequencies, energy and charge are Å, cm^−1^, eV and *e*, respectively. In the left figure, the subunit of the cage-like Ti_12_C_68_ structure is highlighted using blue atoms. Each TiC_13_ subunit is bound to five neighbouring TiC_13_ subunits through different C-C bonds. The pentagon and hexagon are connected by the two kinds different C-C bonds denoted by ${{d}}_{C \mbox{-} C}^{{\rm{1}}}$ and ${{d}}_{C \mbox{-} C}^{{\rm{2}}}$. The pentagons are joined by two kinds C-C bonds presented by ${{d}}_{C \mbox{-} C}^{{\rm{3}}}$ and ${{d}}_{C \mbox{-} C}^{{\rm{4}}}$.

**Figure 2:**
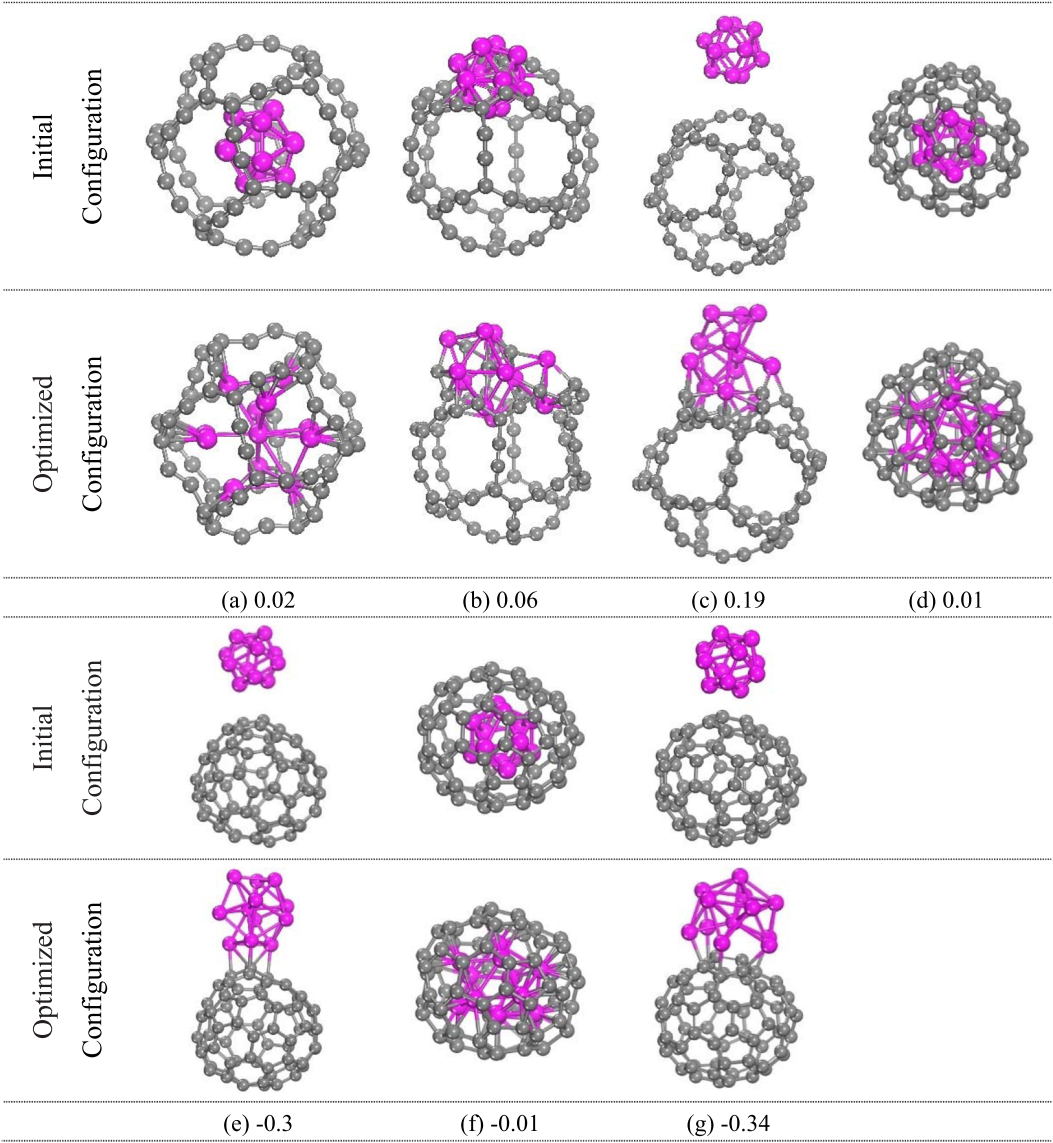
Three representative initial geometric structures and corresponding optimized structures of Ti_12_C_68_ cluster, which are composed by 12 titanium atoms and 68 carbon atoms, as plotted in (**a**–**c**). (**d**–**g**) Isomers of Ti_12_C_68_ cluster are composed of C_68_ fullerene and Ti_12_. The calculated relative averaged atomic binding energies for each isomer, with respect to cage-like Ti_12_C_68_ cluster, is listed underneath each isomer. The unit of averaged binding energies is eV.

In order to search for the ground-state structure of cage-like Ti_12_C_68_ cluster, we have accomplished an extensive search with different initial configurations which are constructed with C_68_ cage and 12 Ti atoms. After relaxing these configurations, it can be found that the Ti atoms prefer to approach the carbon atoms. Three representative initial geometric structures and corresponding optimized structures are plotted in Fig. 2a–c. Note that these structures are larger in averaged atomic binding energy than the cage-like molecule by 0.02, 0.06 and 0.19 eV, respectively. In order to further compare the stability of the structure, we also construct isomers which are composed of C_68_ fullerene and Ti_12_. As for C_68_ fullerene, we choose two isomers with *C*_1_ point group plotted in (d) and (e), and *C*_s_ point group plotted in (f) and (g). The shapes of the fully optimized equilibrium structures of isomers are shown in Fig. 2(d–g). Results show that the Ti atoms prefer to be connected to the C atoms. Moreover, the isomer (d) has a 0.01 eV higher averaged atomic binding energy than the cage-like Ti_12_C_68_. (e), (f) and (g) isomers are found to be lower in averaged atomic binding energy than the cage-like molecule by 0.3, 0.01 and 0.34 eV, respectively. Thus, the cage-like Ti_12_C_68_ is a metastable structure.

**Figure 3:**
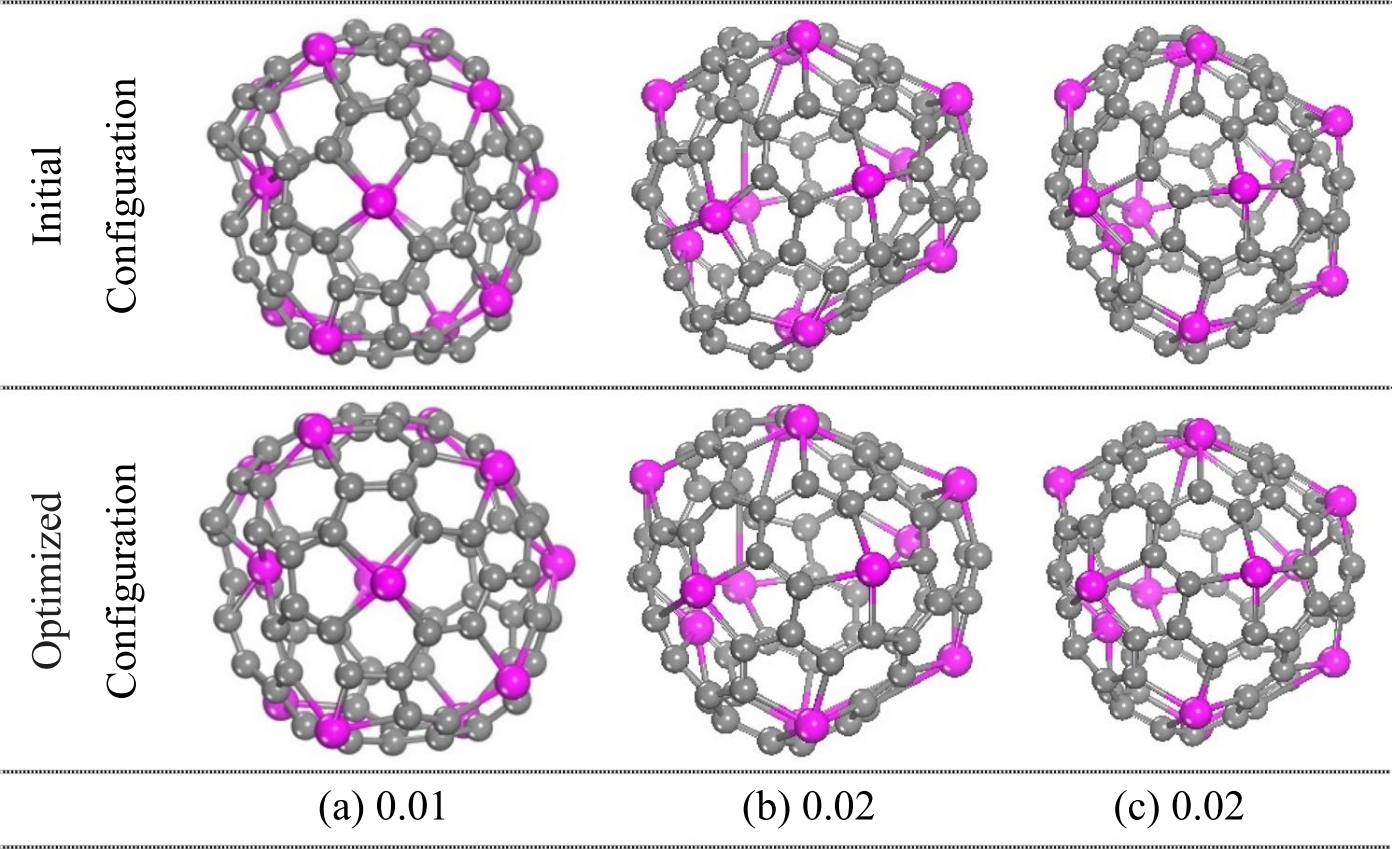
Initial and optimized structures for three isomers of cage-like Ti_12_C_68_ cluster. (**a**), (**b**) and (**c**) are three isomers which are randomly choose from the molecular dynamic simulations at initial temperature of 2000 K and 3000 K, respectively, corresponding to the effective temperature of 952 K and 1343 K. The averaged atomic binding energies in eV, relative to cage-like Ti_12_C_68_ cluster, are listed underneath each isomer.

In addition, in order to further confirm the stability of cage-like Ti_12_C_68_ cluster, we choose randomly three isomers with lower energy from the molecular dynamic simulations in the NVE ensemble at temperatures of 2000 and 3000 K. After energy minimization, the results revealed that the lowest-energy conformation is the cage-like Ti_12_C_68_ cluster in all isomers. The motifs of initial and optimized structures for three isomers are presented in Fig. 3. The calculated relative averaged atomic binding energies (∆*E*_b_) for each isomer, with respect to cage-like Ti_12_C_68_ cluster, is written underneath each isomer.

**Figure 4:**
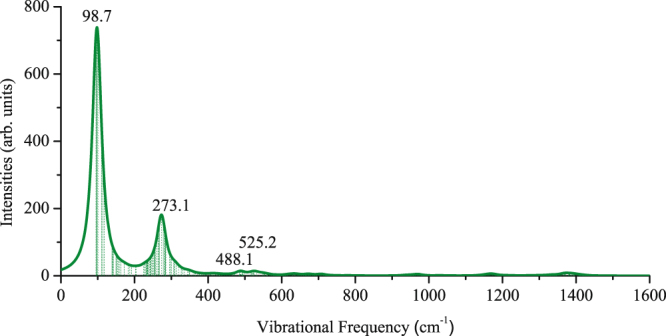
Raman spectrum of cage-like Ti_12_C_68_ cluster. The spectrum is broadened with a Lorentzian of 20.00 cm^−1^.

Then, we evaluated the dynamical stability of the cage-like Ti_12_C_68_ by calculating the vibrational frequencies. Here, the harmonic vibrational frequencies are computed by diagonalizing the mass-weighted second-derivative matrix. There is no imaginary frequencies, which further validates the stability of cage-like Ti_12_C_68_ configuration. The lowest vibrational frequency and highest vibrational frequency corresponds to 95.1 cm^−1^ and 1423.9 cm^−1^, respectively. To provide more information for future experimental identification, we simulated Raman spectrum, which is based on the Raman effect of inelastic scattering of monochromatic light. The energy shift is defined by the vibrational frequency and the proportion of the inelastically scattered light is defined by the spatial derivatives of the macroscopic polarization, technical details are described by Porezag and Pederson^42^. A Raman spectra with the temperature of 300 K and incident light of 488.0 nm was displayed in Fig. 4. We can see clearly that the spectrum has strong peaks at 98.7 and 273.1 cm^−1^ which are due to the Ti-C and C-C stretching modes respectively. More detailed data are described in the Supporting Information (Section I). Therefore, the cage-like Ti_12_C_68_ cluster is kinetically stable.

In what follows, we try to confirm the thermal stability of cage-like Ti_12_C_68_ cluster from *ab initio* NVE MD simulations with the initial temperature at 1400 K, 1800 K, 2000 K, 2200 K and 2400 K, which correspond the effective temperatures of 678 K, 877 K, 950 K, 1072 K and 1139 K. More detail information is presented in the Figs. S1 and S2, where the configurations snapshots of cage-like Ti_12_C_68_ cluster are shown at the 1.25 *ps*, 2.5 *ps*, 3.75 *ps* and 5 *ps* of each MD simulation. After 5 *ps* simulation, the topological structure of cage-like Ti_12_C_68_ cluster was well maintained when the initial temperature is up to 2400 K with the effective temperature of 1139 K. The above observation is an indication of high thermal stability for the cage-like Ti_12_C_68_ cluster.

**Figure 5:**
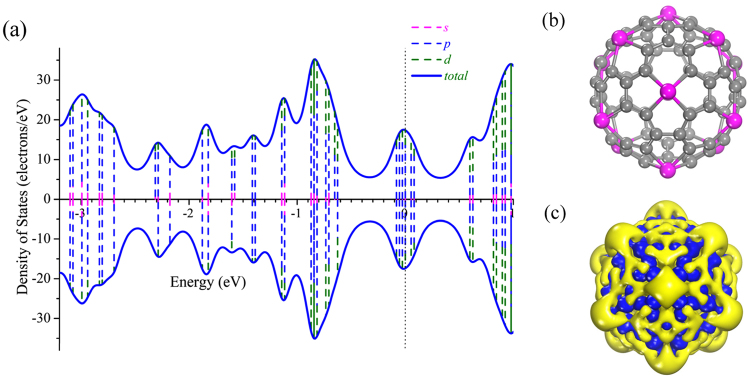
PDOS (**a**), configuration (**b**), and deformation electronic density (**c**) of cage-like Ti_12_C_68_ structure. The Fermi energy is taken as the zero energy. The isosurface for the deformation electron density corresponds to 0.03 *e*/Å^3^.

**Figure 6:**
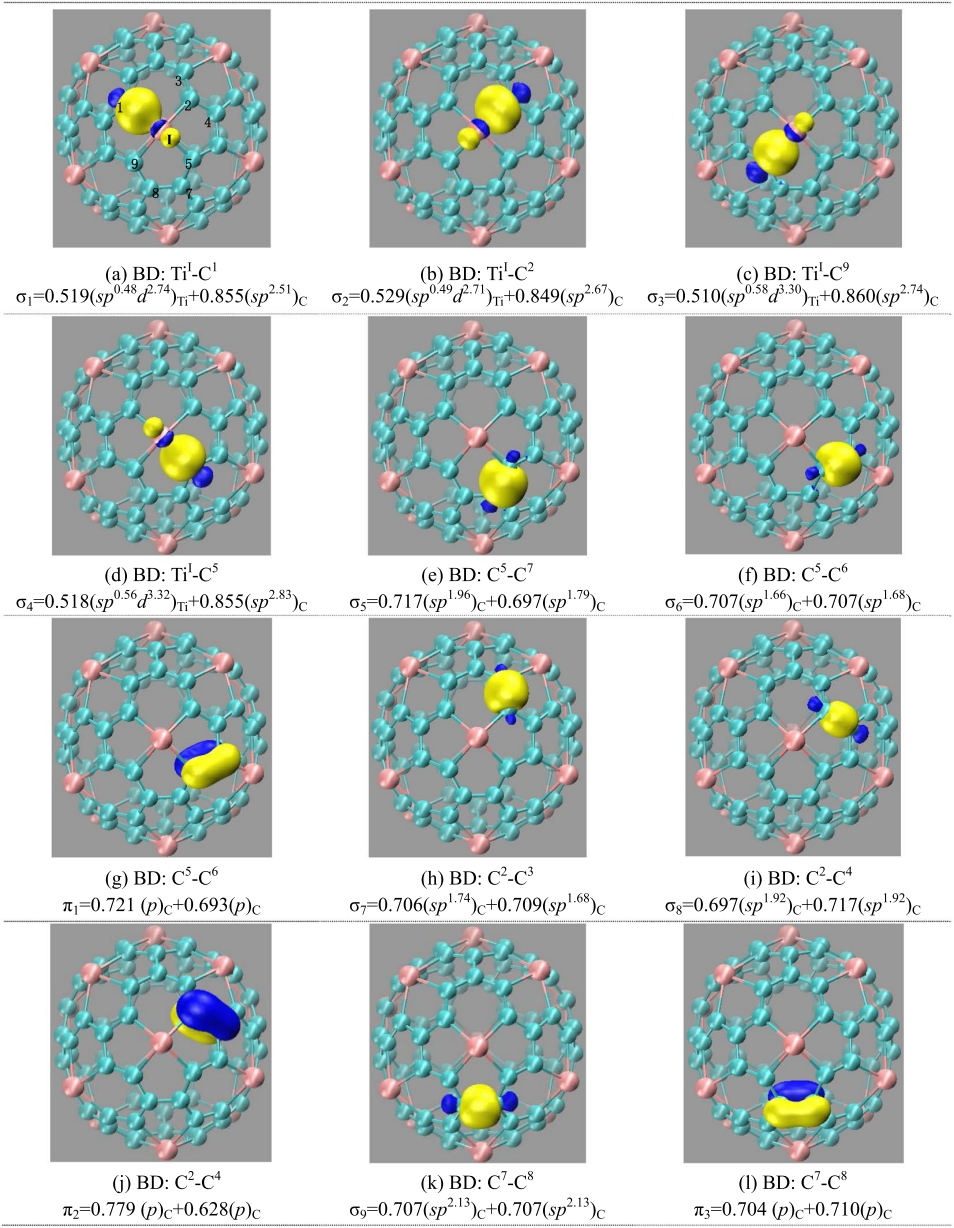
Some typical natural bond orbitals of Ti_12_C_68_ cage. BD is short for 2c-2e bond, and under each structure the detailed hybridization types are provided. All the configurations are viewed by the same orientations.

To get insight into bonding properties of cage-like Ti_12_C_68_ cluster, Fig. 5c displays the deformation electron density, which reveals that electrons are donated from Ti to the C atoms. The removed electrons are mainly from the Ti 3*d* state, and are mainly delocalized to surrounding the four Ti-C bonds. For the Ti atoms, each Ti can connect with four carbon atoms through Ti-C σ bonds. For the carbons that adjacent to Ti atom, each carbon forms σ bonds with neighboring two carbons. Meanwhile, the natural bond orbital (NBO) analysis further elucidates the detailed type of hybridization. Some typical NBO are presented in the Fig. 6. The occupation numbers of natural orbitals are in the range from 1.59 to 1.97 electrons. Here, we label one Ti atom by roman numerals I, C atoms by arabic numerals 1, 2, 3, 4, 5, 6, 7, 8 and 9 as shown in Fig. 6(a). For the Ti^I^ atom, four lobes of *d* orbit form four σ bonds with the neighboring four carbons (C^1^, C^2^, C^5^ and C^9^) atoms, as plotted Fig. 6(a–d). In the σ_4_(Ti^I^-C^5^) bond, it is a two-center two-electron (2c-2e) σ bond with the occupation numbers of 1.87 electrons in hexagon, in which the 26.85% and 73.15% of occupation numbers are situated on the Ti^I^ atom and C^5^ atom, respectively. The bond orbital of the Ti^I^ atom mainly comes from the *d* orbital, and for the C^5^ atom, the hybrids of *sp*^2.83^ are a distorted *sp*^3^ hybridization. In addition, C^5^ atom form two σ (σ_5_ and σ_6_) bonds and a π_1_ bond with two neighbor carbons shown in the Fig. 6(e–g), demonstrating the distortion *sp*^2^ hybridization character of C^5^ atom. For the σ_2_ (Ti^I^-C^2^) bond comes from the hybridization of *sp*^0.49^*d*^2.71^ on Ti^I^ atom and *sp*^2.67^ on the C^2^ carbon in the hexagon as plotted in Fig. 6(b). It is noted that C^2^ atom link the neighbor C^3^ atom and C^4^ atom by σ bonds plotted in Fig. 6(h) and (i), and a π_2_ bond which is formed by the hybridization between *p* orbital of C^2^ atom and *p* orbital of C^4^ atom, as shown in Fig. 6(j). In addition, two identical pentagons are connected by the C^7^-C^8^ bond, from the Fig. 6(k) and (l), it can be seen that C^7^ atom form σ bond and π_2_ bond with the neighboring C^8^ atom. For the all σ(C-C) bonds, it can be found that major contribution comes from *sp*^2^-like hybridization of C atom.

**Figure 7:**
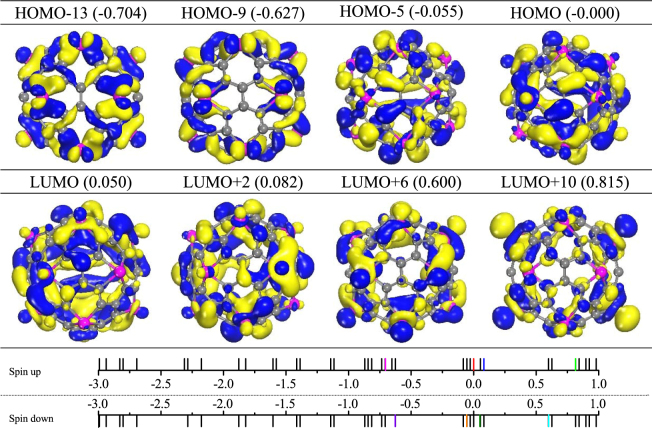
Frontier molecular orbitals for Ti_12_C_68_ cage. The locations of the above molecular orbitals are marked by different color lines, ie. HOMO: red, HOMO−13: magenta, LUMO + 2: blue, LUMO + 10: green, LUMO: olive, HOMO−5: orange, HOMO−9: violet and LUMO + 6: cyan. The isovalues are 0.015 *e*/Å^3^. The eigenvalues are given in brackets with the unit of eV.

Finally, to investigate the electronic structures of cage-like Ti_12_C_68_ cluster. We plotted the projected density of states (PDOS) in Fig. 5a. As can be seen from Fig. 5a, the HOMO and LUMO orbitals show strong *p*-*d* hybridization. The region of 0.6 eV to 1.0 eV and below −0.6 eV, there is *sp*-*d* hybridization character. Furthermore, the plots of frontier orbitals, including HOMO, HOMO−5, HOMO-9, HOMO−13, LUMO, LUMO + 2, LUMO + 6, and LUMO + 10 are given in Fig. 7. As seen from Fig. 7, π bonds form between carbons (for example, HOMO and HOMO−5), which is crucial for stabilizing the structures of cage-like Ti_12_C_68_ cluster. For the HOMO, LUMO, LUMO + 2, LUMO + 6, and LUMO + 10 orbitals, the ${d}_{{z}^{2}}$-like orbitals and other *d*_xy_-like (*d*_xy_, *d*_yz_, *d*_xz_) orbitals characteristic appear on the Ti atoms. Especially for LUMO + 6, and LUMO + 10, orbitals characteristic mainly focus on the Ti atoms. However, HOMO-5, HOMO-9 and HOMO-13 orbitals display sole *d*_xy_-like orbital characteristics on the Ti atoms. While all the given frontier orbitals on C atoms mainly exhibit the *p* orbital character. The above observations are in concordance with the natural bond orbital analysis.

## Conclusions

In summary, we have discovered a stable cage-like Ti_12_C_68_ cluster with *T*_*h*_ symmetry. The stability of cage-like Ti_12_C_68_ cluster has been confirmed using the molecular dynamics simulations and the vibrational frequency analysis. Meanwhile, this cluster is found to have a relatively small HOMO-LUMO gap, which suggests strong chemical activity. More importantly, the density of states and frontier orbitals distributions show remarkable orbital hybridization. The natural bond orbital analysis provides a detailed description including the bond-type of hybridization and occupancy numbers, results show that four lobes of *d* orbit for each Ti atom form four σ bonds with the neighboring four carbons atoms in each TiC_13_ subunit playing a significant role in the structural stability. Moreover, the calculated Raman spectrum of cage-like Ti_12_C_68_ should provide more information for future experimental observations and synthesis. If this molecule can be prepared in a future experiment, it would enrich the species of hollow metal carbide clusters or analogues of metallo-carbohedrenes.

## Computational details

All theoretical calculations are on the basis of the density functional theory (DFT), which begins with a theorem by Hohenberg and Kohn^43^, later generalized by levy^44^, and in which all ground-state properties are functionals of the charge density. For the considered system, except kinetic energy and the classical electrostatic energy, it includes all many-body contributions to the total energy, in particular, the exchange and correlation energies. The exchange-correlation energy, requires some approximation for this method to be computationally tractable. A popular and good approximation is the gradient-corrected approximation (GGA), which can provide a considerable increase in the accuracy of predicted energies and structures as to the local density approximation (LDA). In our calculations, three different exchange-correlation functionals, the Perdew-Burke-Ernzerhof correlation (PBE)^45^, Perdew-Wang (PW91)^46^, and Becke exchange plus Lee-Yang-Parr correlation (BLYP)^47,48^, were employed. For the basis functions, the double numerical including polarization (DNP)^49^ were utilized with the best accuracy and highest cost. For C atom, it is 1s2s2p2s′2p′3d, and it is 1s2s2p3s3p3d4s3d′4s′4p for Ti atom. All geometry optimizations of cage-like Ti_12_C_68_ clusters were performed using spin-unrestricted and symmetry-unconstrained.

*Ab initio* molecular dynamics (MD) simulations, the most natural method of performing equilibrium statistical-mechanical calculations, was selected to evaluate the thermal stability of Ti_12_C_68_ cage. The constant energy, constant volume ensemble (NVE), also known as the microcanonical ensemble, was used. In the NVE ensemble, the total time was set to be 5 *p*s, with each step taking 1.0 *f*s at initial temperature of 1400 K, 1800 K, 2000 K, 2200 K and 2400 K. The natural bond orbital (NBO), that describe the Lewis-like molecular bonding pattern of electron pairs in optimally compact form, is analyzed at the LANL2DZ basis set in Gaussian 09 package^50^.

## Electronic supplementary material

Supplementary Information
