# Supplementary material for: Ti12C68: A stable Th-symmetry hollow cage
Source: Sci Rep. 2018 Mar 8;8:4167. doi: 10.1038/s41598-018-22381-y (PMC5843587; doi:10.1038/s41598-018-22381-y)
Supplement: Supplementary file 1 — Supplementary Information [file 41598_2018_22381_MOESM1_ESM.pdf]

Supplementary information for

$Ti_{12}C_{68}$: A T_h-symmetry hollow cage

Ling-Yan Ai,[1,2] Hui-Yan Zhao[1], Hong-Man Ma[1], Jing Wang,[1] Ying Liu[1,3,*]

[1]*Department of Physics and Hebei Advanced Thin Film Laboratory, Hebei Normal University, Shijiazhuang 050024, Hebei, China.*

[2]*North China University of Science and Technology, Tangshan 063000, China.*

[3]*National Key Laboratory for Materials Simulation and Design, Beijing 100083, China.*

Section I. Raman spectrum data

Tab. S1. The calculated frequencies, Raman activities and intensities of cage-like $T_{12}C_{68}$ cluster. The intensities are broadened with a Lorentzian of 20.00 cm^{-1}, temperature of 300 K and incident light of 488.0 nm.

Frequency (cm^{-1})	Raman activity(Å^4)	Raman intensity	Frequency (cm^{-1})	Raman activity(Å^4)	Raman intensity
95.1	50.015	150.394	583.1	0.057	0.010
96.7	51.549	150.404	584.2	0.082	0.014
98.2	52.273	148.340	605.7	0.044	0.007
98.7	53.173	149.520	615.4	0.915	0.148
100.7	54.337	147.376	620.1	7.712	1.237
110.0	0.550	1.274	623.5	0.546	0.087
113.6	1.030	2.253	628.9	8.092	1.274
117.6	0.080	0.165	630.0	1.368	0.215
138.6	0.025	0.039	636.0	8.357	1.297
139.5	0.032	0.050	638.8	9.459	1.460
140.3	0.013	0.020	641.9	0.171	0.026
142.3	0.028	0.042	645.3	0.319	0.049
142.3	0.048	0.070	646.4	0.209	0.032
149.6	0.024	0.032	647.5	0.300	0.045
151.0	0.203	0.270	664.2	0.151	0.022
153.4	0.218	0.282	664.8	0.059	0.009
157.1	0.206	0.256	666.5	0.493	0.072
158.2	0.087	0.106	667.5	0.769	0.112
162.8	0.319	0.373	669.9	4.589	0.667
171.9	4.369	4.670	670.9	2.749	0.399
183.1	0.242	0.233	672.5	3.681	0.532
187.0	0.101	0.094	673.7	9.611	1.387
191.7	0.498	0.444	675.3	0.527	0.076
202.3	0.027	0.022	676.5	0.533	0.076
204.1	3.189	2.565	678.1	0.334	0.048
224.5	1.261	0.870	679.6	1.230	0.175
227.7	3.026	2.041	683.9	0.491	0.070
228.8	3.885	2.600	688.2	0.386	0.054
231.7	2.477	1.625	706.1	8.626	1.173

* Correspondence author. E-mail: yliu@hebtu.edu.cn

233.3	1.797	1.166
234.1	0.576	1.166
235.4	0.051	0.372
236.2	0.050	0.033
237.4	0.661	0.032
241.2	0.559	0.417
242.2	1.437	0.344
244.4	1.587	0.879
244.6	1.393	0.957
246.5	0.342	0.839
249.1	0.314	0.203
250.7	0.524	0.184
254.1	3.968	0.304
254.9	0.251	2.250
254.9	0.533	0.142
256.2	0.382	0.301
260.5	0.247	0.214
264.4	0.213	0.135
265.5	0.065	0.114
268.3	0.345	0.035
273.0	319.118	0.180
273.2	4.431	161.934
277.9	4.787	2.246
281.3	1.751	2.363
282.3	5.032	0.849
283.7	2.958	2.426
284.2	1.008	1.415
285.5	0.479	0.481
294.9	0.033	0.015
298.8	0.018	0.008
299.8	0.014	0.006
306.6	5.664	2.409
307.4	5.159	2.186
308.8	6.625	2.788
312.9	14.326	5.912
319.6	1.152	0.461
326.9	0.403	0.156
331.1	1.123	0.426
335.7	1.133	0.421
344.5	4.270	1.529
345.5	2.590	0.923
348.9	4.486	1.577
350.0	3.250	1.137
359.4	0.202	0.068
363.4	0.091	0.030
365.8	0.078	0.026
707.3	10.647	1.444
708.4	9.341	1.265
741.5	0.142	0.018
743.8	0.034	0.004
776.5	0.541	0.065
779.9	1.032	0.124
782.2	0.296	0.035
784.2	0.109	0.013
785.5	0.916	0.109
786.1	1.418	0.169
921.6	0.621	0.061
932.8	0.821	0.079
935.7	1.620	0.155
936.5	4.735	0.452
937.1	0.346	0.033
939.2	4.497	0.428
946.5	2.481	0.234
948.2	1.345	0.127
950.2	0.281	0.026
952.8	1.574	0.147
954.9	4.958	0.462
961.0	0.411	0.038
963.8	1.212	0.112
970.0	43.933	4.019
1037.3	0.137	0.012
1038.9	1.075	0.090
1040.4	2.592	0.217
1041.7	1.530	0.128
1043.1	1.692	0.141
1046.1	2.422	0.202
1133.6	2.741	0.206
1145.1	5.029	0.374
1153.1	11.439	0.843
1158.9	5.781	0.423
1163.2	10.714	0.781
1164.6	20.777	1.512
1171.3	1.520	0.110
1172.3	24.755	1.787
1176.4	16.060	1.154
1180.4	7.283	0.521
1200.7	0.418	0.029
1203.8	1.503	0.105
1209.1	4.239	0.294
1217.6	1.309	0.090
1219.4	1.493	0.102
1223.6	2.246	0.154

372.1	0.085	0.027	1236.4	0.694	0.047
376.5	0.011	0.003	1237.8	1.136	0.077
378.4	0.048	0.015	1254.7	0.224	0.015
382.5	0.037	0.011	1262.2	0.890	0.058
392.5	0.009	0.003	1265.3	1.467	0.096
394.5	0.026	0.008	1273.6	1.565	0.102
407.5	2.694	0.758	1273.8	1.276	0.083
411.7	3.211	0.891	1276.1	1.254	0.081
420.3	2.752	0.742	1280.2	2.468	0.159
426.0	2.562	0.678	1283.5	2.217	0.143
427.0	1.022	0.270	1289.1	1.821	0.117
429.2	0.364	0.095	1301.4	4.950	0.313
431.2	0.069	0.018	1308.6	4.209	0.264
436.6	0.334	0.085	1317.6	1.966	0.122
438.4	0.779	0.198	1322.0	2.874	0.178
461.6	0.063	0.015	1330.5	4.068	0.250
470.9	0.099	0.023	1332.7	18.937	1.161
471.8	0.095	0.022	1337.6	2.222	0.136
485.4	20.637	4.567	1342.2	2.003	0.122
487.2	1.255	0.276	1343.7	1.303	0.079
488.8	1.058	0.232	1346.0	3.100	0.188
489.6	24.747	5.413	1354.0	6.996	0.420
492.3	0.597	0.130	1358.1	2.257	0.135
494.0	0.268	0.058	1364.4	5.905	0.351
495.4	0.086	0.019	1368.3	59.356	3.516
498.4	0.050	0.011	1369.4	1.227	0.073
516.7	0.710	0.144	1372.1	22.323	1.317
518.7	0.978	0.198	1379.3	1.368	0.080
520.1	8.321	1.679	1380.2	13.762	0.806
522.9	14.403	2.885	1381.4	3.380	0.198
524.9	12.339	2.459	1383.3	24.744	1.445
531.7	17.641	3.457	1386.7	9.447	0.550
548.8	8.293	1.558	1392.2	15.935	0.923
550.9	5.498	1.028	1394.6	14.548	0.841
552.5	4.238	0.789	1395.1	3.448	0.199
553.1	1.812	0.337	1398.2	7.366	0.424
559.0	1.213	0.222	1400.3	5.377	0.309
560.0	2.779	0.509	1403.4	6.830	0.391
561.5	0.071	0.013	1408.6	6.427	0.367
569.5	0.030	0.005	1409.5	6.038	0.344
578.6	0.209	0.037	1418.1	9.564	0.541
579.7	0.150	0.026	1423.9	6.364	0.358

Section II. *Ab* initio molecular dynamics

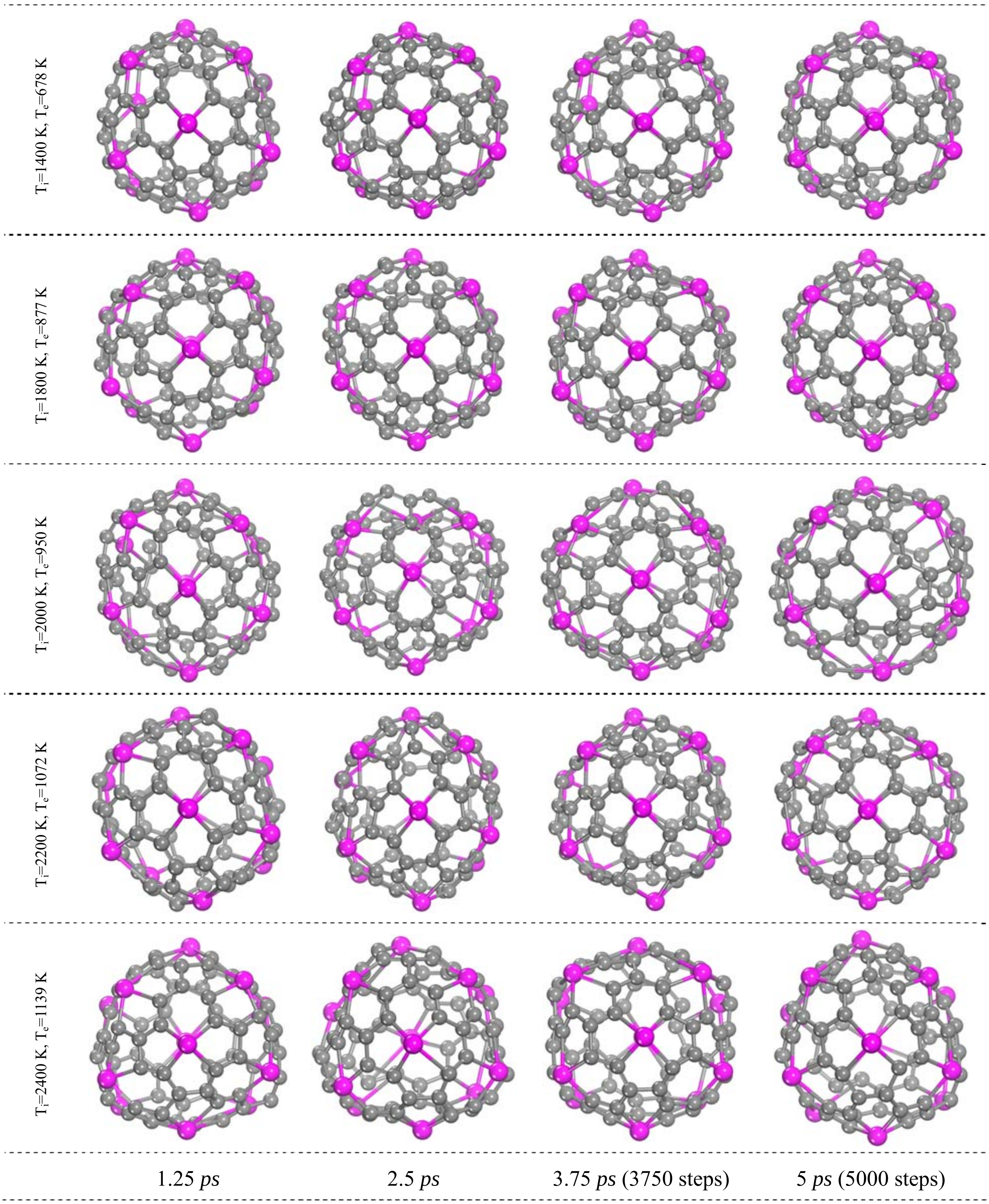

Fig. S1. NVE MD simulation of $Ti_{12}C_{68}$ cluster at initial temperature of 1400 K, 1800 K, 2000 K, 2200 K and 2400 K. T_i and T_e denote the initial temperature and effective temperature, respectively. The snap shots of $Ti_{12}C_{68}$ cluster are shown at the 1.25 *ps*, 2.5 *ps*, 3.75 *ps* and 5 *ps*.

Section III. Comparison of calculated results based on three levels of optimization

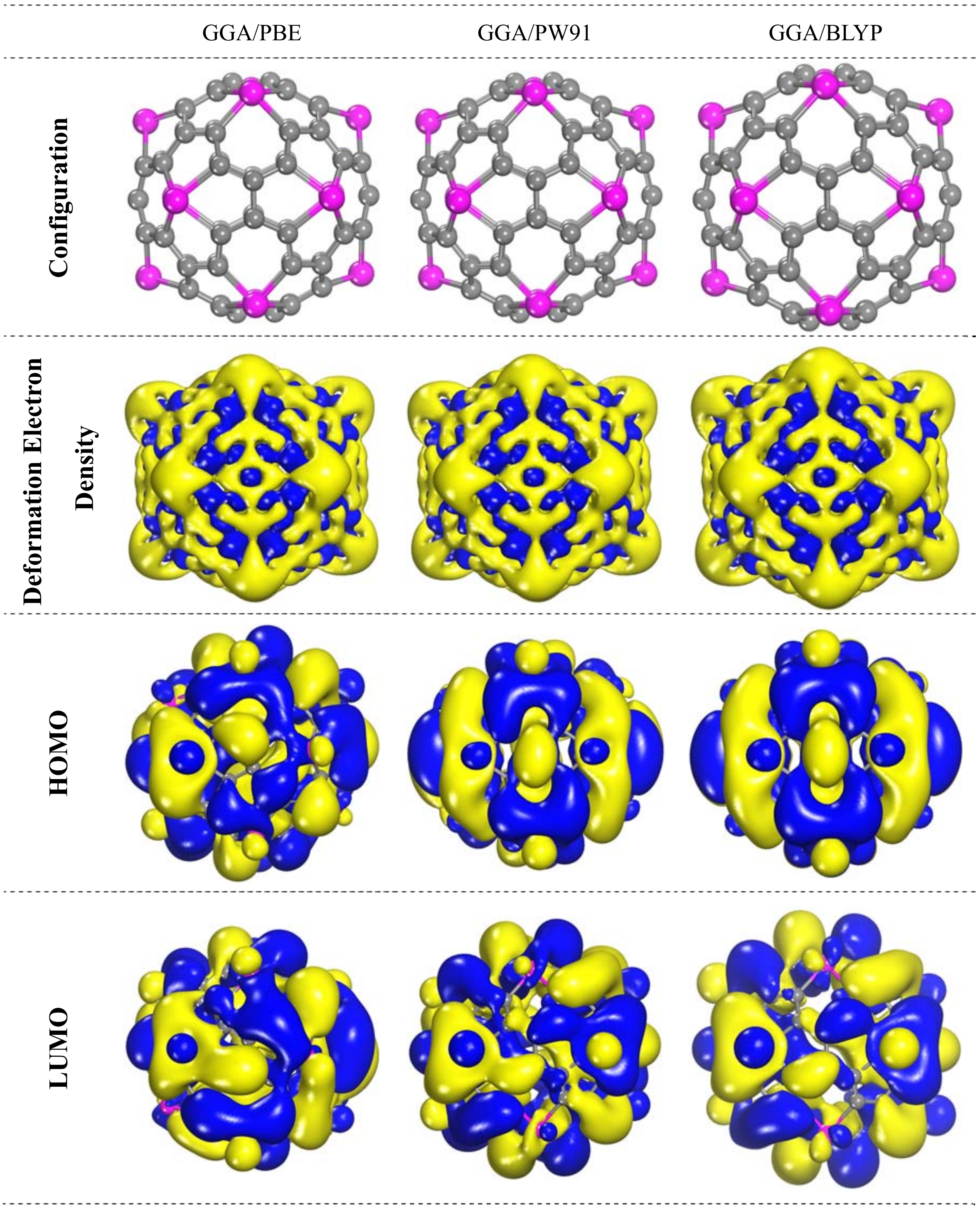

Fig. S2. The configurations, deformation electron densities, HOMO and LUMO orbitals are shown from top to bottom at the GGA/PBE, GGA/PW91 and GGA/BLYP levels. Three optimized structures and deformation electron densities are given with similar characteristics. For the three LUMO orbitals, they are also similar. However, the HOMO orbital at the GGA/PW91 level is same with the orbital using BLYP exchange-correlation functionals. The isosurfaces are 0.03 $e/\mathrm{A}^3$and 0.005 e/A^3 for the deformation electron density and orbital, respectively.
